# Can grammatical morphemes be taught? Evidence of gestures influencing second language procedural learning in middle childhood

**DOI:** 10.1371/journal.pone.0280543

**Published:** 2023-02-01

**Authors:** Natasha Janzen Ulbricht

**Affiliations:** Department of Philosophy and Humanities, Freie Universität Berlin, Berlin, Germany; University of Hull, UNITED KINGDOM

## Abstract

What kind of practice makes perfect when children learn to use grammatical morphemes in a second language? Gestures are communicative hand and arm movements which teachers naturally employ as a teaching tool in the classroom. Gesture theory has proposed that gestures package information and previous studies suggest their value for teaching specific items, such as words, as well as abstract systems, such as language. There is broad consensus that implicit learning mechanisms in children are more developed than explicit ones and that everyday use of grammar is implicit and entails developing implicit knowledge. However, while many learners have difficulties acquiring new morpho-syntactic structures, such as the plural{-s} and 3^rd^ person possessive {-s} in English, research on gesture and syntax in middle childhood remains rare. The present study (N = 19) was conducted to better understand if gestures which embody grammatical morphemes during instruction can contribute to procedural learning. Using a novel task, the gesture speeded fragment completion task, our behavioral results show a decrease in mean response times after instruction in the test condition utilizing syntactically specific gestures. This increase in procedural learning suggests that learners in this age group can benefit from embodied instruction in the classroom which visually differentiates between grammatical morphemes which differ in meaning but sound the same.

## Introduction

The process of learning a second language is complex, dynamic and often nonlinear [1]. Behavioral and neuroscientific studies suggest that the neural representations of words consist of complex multimodal networks represented in sensory and motor brain areas in an experience-dependent way [2,3]. Thus it is unsurprising that gesture has been shown to influence how we understand complex language [4] and learn abstract concepts [5]. Building on the idea that cognitive representations are grounded or embodied via perception and action, this study focuses on an important aspect of second language (L2) syntax and investigates how to facilitate teaching two important L2 grammatical morphemes through gestures for the plural{-s} and the 3^rd^ person possessive {-s} in English.

Gestures are communicative hand and arm movements which embody emotions, intentions and thoughts [6–8]. There is much research providing empirical support for the role of gestures in L2 learning for aspects such as speech comprehension [9,10], word memorization [11,12] and pronunciation [13–17]. Not unexpected, teachers naturally use gestures as a teaching tool in the classroom and previous studies suggest their value for L2 instruction. At the same time, research also shows that adding gesture does not automatically improve learning outcomes [18–22] leading researchers to call for more specific predictions about which gestures support learning and when these gestures will be helpful [23–25]. Related arguments from many areas of cognitive science have highlighted that it is important to examine the specific types of interaction between syntax and semantics and grounding that lead to understanding [26]. (See [27] for a meta-analysis on when gesture benefits listener comprehension. See also [28] for an overview related to the embodiment of syntax and grammar in the brain).

Gesture researchers have proposed the Gesture-for-Conceptualization Hypothesis (GfCH) which states that gestures can schematize information and conceptually link hand movements not only to speaking, but also more generally to thinking itself [29]. Because observing gesture triggers semantic processing [30,31] it is conceivable that gestures linked to L2 grammatical morphemes could help children learn. However, gesture research suggests that gestures must be semantically related to words in order to support long term memory [32]. It is further hypothesized that it is because gesture activates visual representations of concrete concepts that it facilitates learning [33]. This raises the question of what exactly gestures for syntactical morphemes would map onto. Following this line of argumentation, L2 syntax, lacking an established concrete visual referent, may be too abstract and as such gestures would not help.

### Learning and memorization

A leading tenet of neurobiological theory on learning and memory is that two at least partially independent neural systems, the declarative and procedural memory systems, underlie learning, representing and applying relevant knowledge [34–36]. Declarative knowledge, associated with learning and using novel events and facts can be quickly learned, but is slower to use, necessitates more cognitive resources than procedural knowledge, and may be rapidly degraded. Procedural knowledge, in contrast, has been implicated in skill learning and habits. Procedural knowledge requires a critical amount of practice and time and is sometimes conceptualized as implicit learning. Through the procedural memory system knowledge with a complex structure can be acquired to a large degree independently of awareness of both the process and product of acquisition [37]. Applied to L2 learning, declarative knowledge includes knowledge of morphology, as well as grammar rules and is processed slowly. Procedural knowledge is quickly processed in parallel with other cognitive processes and thus places less of a burden on working memory [38]. In this experiment, as in others, participant characteristics, such as the role of knowledge about L1 grammatical morphology, influence individual L2 learning outcomes. According to Boas and Höder [39], language contact can be seen as the normal state of languages, speaker groups and individual speakers. Although not many families reported that their children had an L2 other than the language of instruction, which was German, classroom observation suggests that diverse linguistic resources sometimes played a role in normal classroom interaction and thus it is possible that they were sometimes used during L2 learning. These and other confounding variables were dealt with by utilizing a within participant design, meaning that children in the two different test conditions were compared to themselves and thus cannot influence experimental outcomes.

### Explicit and implicit learning

It is known that implicit learning mechanisms in children are more mature than explicit ones and there is broad consensus that everyday use of grammar is implicit. While it is implicit knowledge which enables both L1 and L2 learners to use language productively [40], it is not clear which memory system is directly involved in any given linguistic task [23,24]. L2 related research provides evidence that declarative knowledge may be converted into procedural knowledge (proceduralization of declarative knowledge) and procedural (implicit) knowledge may be converted into declarative knowledge as a result of experience [41].

### Grammar and syntax learning

Perhaps unsurprisingly the subject of if and how grammar should be taught has long been debated [42] and linguists have not only stated that syntax should not be taught, but even more controversially, that it cannot be taught [43]. This is obviously not the case [32,33]. While there is an obvious difference between advising against formal instruction because there is a better way and stating that syntax cannot be taught, this controversy has continued. Many learners fail to master appropriate L2 use and many teachers tend to be skeptical about their grammar instruction [44].

Syntax has been defined as the study of the organization and interrelation of grammatical elements [45]. In the present study, to better understand if gestures which embody grammatical morphemes support procedural learning for syntax, we explore the impact of gesture on response time. For the purpose of this study, teaching and testing the English L2 plural{-s} and the 3^rd^ person possessive {-s} are useful because while children are frequently taught rules associated with these grammatical morphemes, the concepts are complex [40,46] and procedural learning takes time. In summary, I argue that the plural suffix -s and the -’s clitic marking the genitive case are important because they encode grammatical categories, are syntactically relevant and are fully productive in that they can be attached regularly to any word of the appropriate class [47] (see also [28]).

### Gestures for thinking and speaking

As previously mentioned, researchers have recently proposed the Gesture-for-Conceptualization Hypothesis (GfCH) stating that gestures schematize information and are conceptually linked to thinking as well as speaking [29]. Observing gestures triggers semantic processing [31,48] and related to L2 learning, gestures could allow linguistic units, such as the plural{-s} to be paired with a hand movement (see also [49]). This stable movement-meaning connection could reduce the need for other aspects of language comprehension and allow the brain to save these cognitive resources for additional information processing, leading to more robust consolidation and better retention [50,51]. Brouwer, Fitz and Hoeks have proposed the term mental representation of what is being communicated (MRC) for the internal representation a listener or reader constructs while comprehending a sentence, story or scene [52] (see also [53]). They specify that MRCs are not only derived directly from linguistic input, but also from inferences made on the basis of logical, causal or pragmatic world knowledge [52]. It follows that if in addition to patterns available in speech, gestures make it easier to retrieve and integrate stored knowledge, this would translate into semantic prediction leading to more efficient mental processing [54].

Along the lines of other situation model theories [55], and relevant to linguistic theory, if meaningful gestures enable learners to update their MRC with less effort and more clarity, learning would be less tied to contextual familiarity and more prone to consolidation. On the other hand, if gestures cannot be mapped onto a meaningful pattern, they would interfere with linguistic processing and language learning. Gesture theory, as outlined in the GfCH, makes predictions about the supportive effects of gestures for learning, but, as the MRC concept suggests, much of the information used to determine meaning is not associated with one lexical item [26], so many questions remain unanswered when it comes to how best to use gestures in language instruction.

### Studies on procedural learning and syntax in middle childhood

While the relationship between gesture and L2 teaching and syntax learning has been examined, few if any studies have practically examined the effect of gesture on procedural learning for L2 syntax in classroom settings with learners of primary school age. This research gap is unfortunate because it is here, in this setting and age group when many learners begin formal second language learning. Quantitative behavioral studies related to procedural learning and syntax in middle childhood are reviewed and summarized in Table 1.

**Table 1:** Previous studies involving procedural learning and syntax with primary age children.

Researchers	Participants	Study Objective
Eghbalzad, Deocampo & Conway (2021) [56]	26 children 8–12 years old	To investigate the relationship between pattern recognition ability, socioeconomic status and language outcomes
Kidd & Arciuli (2016) [57]	68 children 6–8 years old	To examine the role individual differences in a non-linguistic visual task play in predicting syntax comprehension
Lum, Conti-Ramsden, Page & Ullman (2012) [58]	51 children with specific language impairment (SLI) and 51 typically developing children (mean age 10 years)	To test and examine differences in the relationship between measures of working, declarative and procedural memory and the lexical and grammatical abilities of children with and without SLI
Ferman & Karni (2010) [59]	24 participants eight from each age group; 8 year-olds, 12 year-olds and young adults (mean age 21 years)	To investigate age differences in declarative and procedural learning for producing and judging an artificial morphological rule

To shed more light on gesture and L2 learning a recent study investigated the influence of teacher gestures on oral fluency in a diverse group of primary school age children [60]. This experiment implemented two L2 methods of language instruction, one with teacher gestures at the level of morphology, and one with gestures at the sentence level plus the written text. When the teacher gestured at the level of morphology (e.g. it + is + dark + out + there) there was one hand movement for every morpheme. In the case of this example sentence, five gestures were used, because no words are morphologically complex. (Sentences with morphologically complex words (e.g. final + ly + every + one + is + sleep + ing) had more than one gesture per word.) When the teacher gestured at the level of the sentence, there was one hand movement (e.g. it is dark out there) which corresponded with the entire sentence. For the children who learned with gestures at the level of morphology, speech and gesture were the only forms of linguistic input during training. For the children who learned with gestures at the sentence level plus the written text, the first half of the training time was spent reading and learning the written text and the second half of the training time was spent going through the play using the sentence level gestures to memorize the text.

Results from this first experiment showed a difference in long-term fluency gain between the experimental conditions among high and low performers. It was observed while learners with a lower initial speech rate benefitted more from gestures at the level of morphology, those with an initially higher speech rate benefitted more from reading plus sentence-level gestures. This suggests that the initial fluency level of learners is predictive of which type of gesture benefits fluency the most. A follow-up study using the same teaching methods investigated spatial term learning [61]. Here it was found that for these more abstract words, gestured input at the level of the morpheme, as opposed to reading plus gestures at the sentence level, benefitted all learners, regardless of their initial level. Results from these two gesture experiments beg the question where the long-term improvements in learning come from.

### Background on gestures in the experiment

Although gestures have been grouped and named according to many classifications, the term codified gesture simply refers to gestures with meanings stored as a stable link in long-term memory [62]. According to the foreground-background gesture framework [63] codified gestures are foreground gestures and are comparable to an entry in the mental lexicon where a constant hand shape and movement is assigned a stable meaning. Codified gestures can be iconic, such as meaning ‘cat’ when placing fingers on both sides of the mouth to suggest whiskers. On the other hand, codified gestures can also be determined without an obvious concrete form meaning relationship. For example, borrowed from French sign language, one could tap the forehead to create a gesture meaning ‘pourquoi’ for ‘why’. In the classroom when a teacher performs a new gesture, the semantic relationship between movement and learning content must be immediately apparent, otherwise the hand movement may not be understood and must be learned by association. When meaningful gestures are combined with new words, learners may benefit since gestures can be perceptually similar to the object or event being referenced and can thus add semantic information. This, additional embodied semantic information can in turn prime lexical representations [64]. It is important to note that in gesture research there is wide agreement that hand movements can be categorized into different subtypes [29]. Although the gestures used in this study could be categorized in other ways (e.g. [7]), the term codified gestures has been used to emphasize the one-to-one relationship between movement and meaning. At different times research on L2 learning has used different terms for similar movements-meaning pairs sometimes creating new terms, such as Intentional Teaching Gestures [65] or Voice Movement Icons [66] and at others simply referring to gestures (e.g. [24,67]).

### Present study

Vocabulary learning has been the focus of much research on gesture and L2 instruction. These experiments, while crucial, lack the precision necessary to provide guidance on whether gestures might support learning to use grammatical morphemes in context or not. The present study extends this work and reports the results of a three-week experiment that tested the effects of gesture-based instruction on L2 plural{-s} and 3^rd^ person possessive {-s} use in English.

Since it is difficult to directly view the rules and structures a learner has internalized, one possibility to assess learning is to look at performance and production errors [68]. This can be done by providing instruction in one context, such as playing language games in a group, and testing a possible transfer of learning on an individual transfer task, such as the GSF task.

I hypothesize that during second language acquisition gestures can support the mental representation of what is being said (MRC), reducing uncertainty and resulting in semantic prediction which facilitates more efficient language processing. Based on previous unpublished results and in agreement with usage-based models of language acquisition [69] I make no prior claims about one condition, the syntactically specific two gesture condition or the syntactically general one gesture condition, being more efficient than another. Following a repeated-measures design, which quantifies changes over time, a potential gain in procedural learning measured by a decrease in response time was analyzed. This approach is consistent with the premise that meaning is embodied and that learning occurs as a result of collaboration with others in familiar socially constructed settings [70–72], and addresses the following research questions:

1. In the context of a group training in which children use gestures corresponding to the plural s and possessive s, can a long-term gain in L2 procedural learning for the use of these grammatical morphemes be measured on an individual semantic priming transfer task?
2. Can we find evidence that seeing different grammatical morphemes for the plural and possessive s in gesture form results in measurable differences in response time?

Results will add to our general understanding of the mechanisms by which children learn and explore the nuances of when grammatical morphemes in gesture form help.

## Materials and methods

This research used a novel version of the computer-based speeded word fragment completion task [28] I refer to as the gesture speeded fragment completion task. Before and after four hours of group instruction children of one school class between 11 and 12 years old (N = 19) completed phrases, such as the dog’s n_ck (neck) or the dogs pl_y (play) from which one letter was omitted, as quickly as possible. Identical phrases were completed in two conditions, a syntactically specific (two-gesture) condition and a syntactically general (one-gesture) condition. In both conditions each item consisted of viewing the first three morphemes in gesture form (e.g. the + dog + s) followed by a semantically related written fragment (e.g. n_ck (neck)) where response time was measured on completion. Whereas the syntactically specific condition had two ‘s’ gestures; one for possessive and another for plural, the syntactically general condition had only a single ‘s’ gesture for both. All ‘s’ gestures were iconic in that their form corresponded to their sound, but while the syntactically specific gestures visually distinguished between their plural and possessive meanings, the general ‘s’ gesture did not. (See Fig 2 in the Materials and Methods section for a comparison between the possessive, plural, and general ‘s’ gestures).

### Participants

Our study was conducted with a convenience sample of twenty-three learners between the ages of 11 and 12 who attended the same primary school class in urban Germany (*M* = 11 years, *SD* = 0.32, 10 females). In week 1 and 3 children were tested using the gesture speeded fragment completion task (GSF task) where they completed semantically related phrases such as *the cat’s t_il* (tail) or *the cats lo_k* (look) to measure initial learning and retention. In week 2 of the experiment, children received instruction for a total of four hours over four days. Of the grade 6 children, 2 identified an L1 other than German as their primary home language. All children reported having previously learned English.

### Ethics statement

The experimental procedure was approved by the city department of education as well as the school leadership before the study began. Parents read an information sheet containing general information about the experiment and data treatment. All children who participated submitted written consent from their parents prior to the study and agreed to participate. After data analysis was complete the children were debriefed about the experiment and had the possibility to ask questions.

### Design

This study employed a within participant pretest-posttest design with response time as the main dependent variable and condition (syntactically specific vs syntactically general), and time (session 1 vs session 2) as independent variables. In week 1 and week 3, before and after training instruction children were individually tested. Because the test items were randomized in their order and the order of which experimental condition came first was counterbalanced across participants, session tests consisted of different versions of the same test [73,74].

### Materials

This experiment investigates the role which gestures embodying grammatical morphemes can play in the acquisition of procedural knowledge for L2 phrases across multiple learning sessions. The study consisted of group instruction and the GSF task, a task designed to examine possible semantic priming effects for perception of these grammatical morphemes in gesture form. Training sessions focused on learning and using codified gestures for 32 simple English phrases such as *the boy’s t-shirt* or *the cats look*. Half of the phrases followed a [NOUN + POS-S + NOUN] pattern and half followed a [NOUN + PL-S + VERB] pattern. The experiment proper began with a group warm-up familiarization phase. This was followed by individual GSF pretests. This was followed by group instruction and, finally, by individual GSF posttests. Tasks are described one by one below in the order in which children encountered them.

### Warm-up training

**Fig 1:**
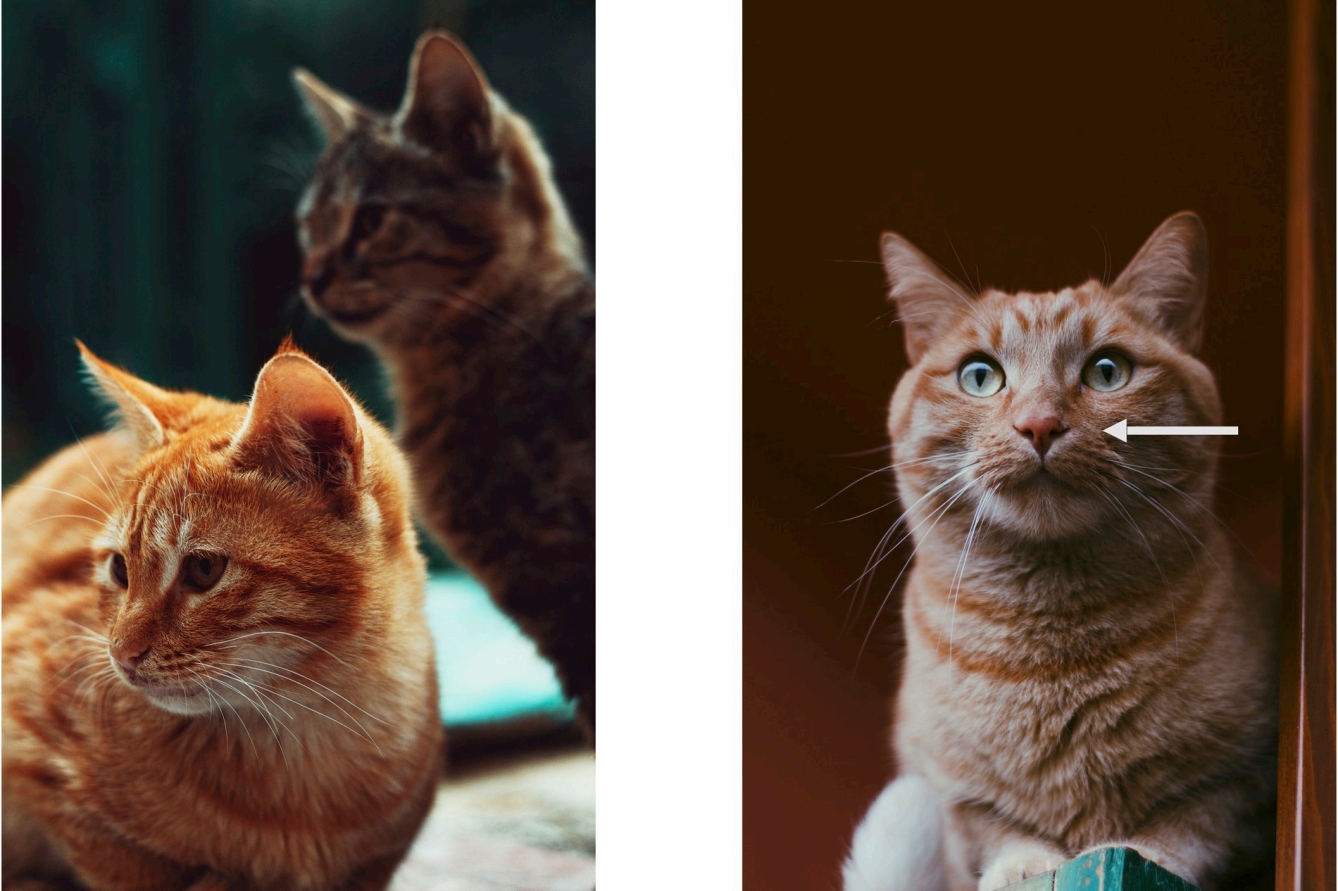
Sample pictures used in instruction in the cat training phases. Pictures correspond to *the cats look* and *the cat’s nose*. Source of all images in the learning procedure: https://unsplash.com.

During the warm-up training children were introduced to the 40 nouns and verbs used in the experiment. A list was presented and discussed to clarify less familiar words. Instruction then paired the written words with gestures and finally with pictures. This sequence served to familiarize children with the word-gesture pairs and to avoid children mapping the gesture for *boy* to unintended objects in the pictures (e.g. the t-shirt the boy was wearing) had they seen the pictures first. Two pictures associated with the *cat* phrases can be seen in Fig 1. Note that during the warm-up training, where the purpose was to reinforce word meaning (e.g. for the word *crash*), some of the pictures used differed slightly from those later used during instruction to reinforce phrase meaning (e.g. *the car’s crash*). During the warm-up training, for example, the picture paired with *crash* showed only one car crashing into a wall.

### Gesture speeded fragment completion task

This experiment used a novel version of the speeded word fragment completion task [75] called the gesture speeded fragment completion task. Semantic priming is the finding that the processing of a target (e.g. a picture, word or sound) is enhanced when preceded by a semantically related prime (e.g. a picture, word or sound) relative to an unrelated prime. Aspects of word meanings are reflected in the topography of brain activation and priming corresponds to a transfer of activation between two lexical representations and can reveal the nature of the connection between the two units or the existence of shared representations [76]. Priming is used to study semantic access in the mental lexicon and much current neuroscientific research focuses on prediction in perception and action. This can be summarized as follows: “When perceiving a series of events, the item occurring next can frequently be anticipated some time before it occurs, and similarly, in performing a series of motor acts, the next-following one is typically processed before its onset” [54]. While different word fragment completion tasks are used to examine semantic priming, the general idea is that participants are presented with words from which one or more letters have been omitted and while participants fill in the gap their response time is measured. The main dependent variable in such experiments is response time. Building on this general principle, Heyman and colleagues created a task using stimuli with only one blank space, where stimuli have only one correct completion and the missing letter is always a vowel [75]. Because these qualities make the task engaging and allowed for a fine-grained investigation of semantic activation in the past, it was adapted it for using with L2 learners.

**Fig 2:**
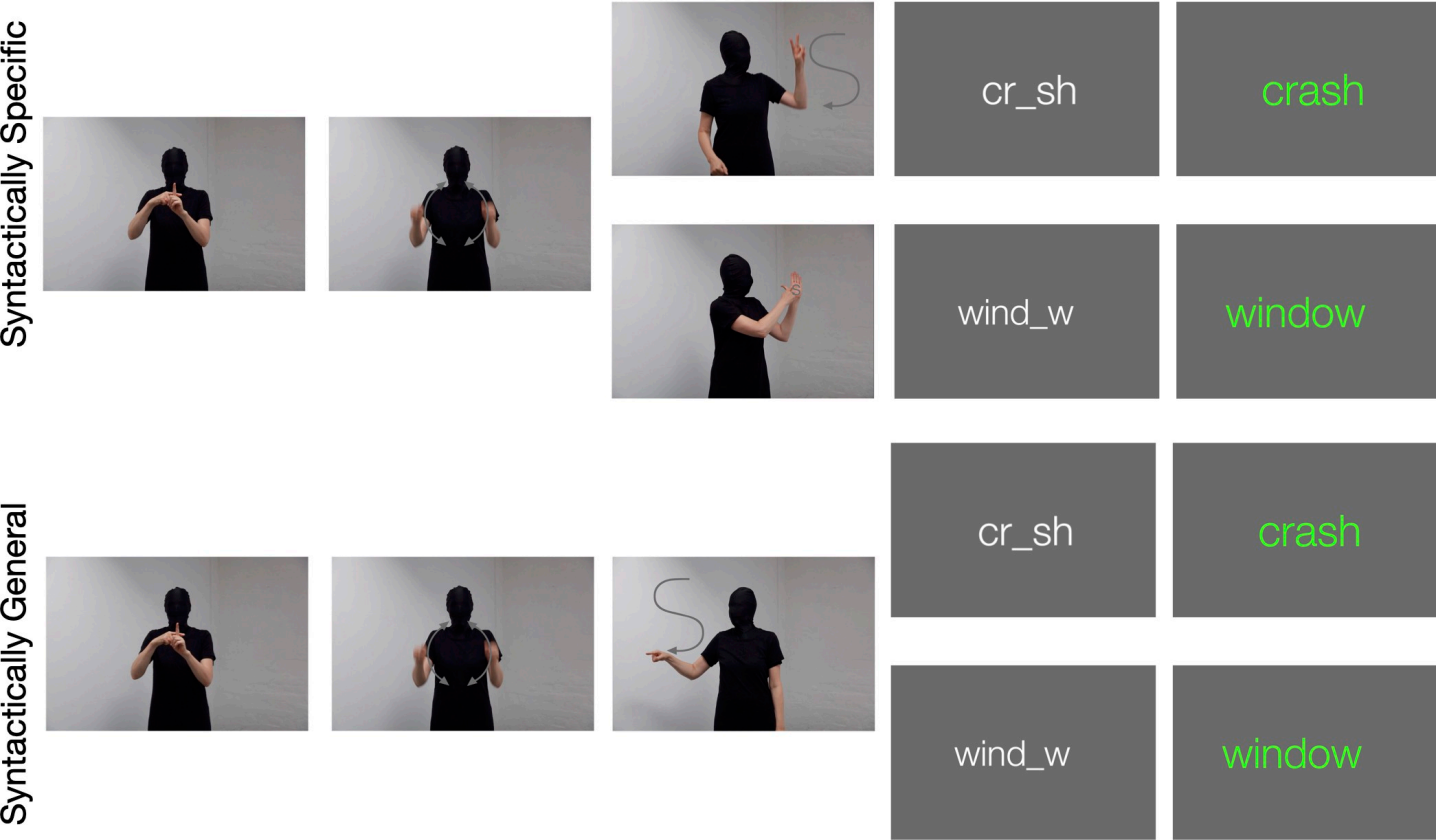
Schematic comparison of gestures corresponding to items *the cars crash* and *the car’s window* in both conditions. A link to demonstration videos in both conditions can be found in the S1 Appendix.

The gesture speeded fragment completion task exploits the homophone-like stimuli of *car’s* vs *cars* or *dog’s* vs *dogs* where a phrase such as *the cars crash* is represented by three morphemes in gesture (the + car + s) followed by a semantically related word fragment (cr_sh (crash)) which measures the response time necessary to complete the fragment. Each test is comprised of 32 items (see S1 Appendix) in two conditions. In Fig 2 the upper sequences represent the syntactically specific (two-gesture) condition and the lower sequences the syntactically general (one-gesture) condition.

As previously mentioned, ‘s’ gestures in both conditions were iconic in that their form corresponded to their sound, but while the syntactically specific gestures in the upper half of Fig 2 made a visual distinction between their plural and possessive meanings, the general ‘s’ gesture in the lower half did not.

Children were tested after the warm-up training (pretest) and after instruction (posttest). Conditions were blocked, meaning that an individual child had all 32 items in randomized order with either the syntactically specific or the syntactically general condition first. A testing session lasted between 10–15 minutes including a short break between the two conditions. PsychoPy Experiment Builder (v3.1.2) was used to create and run the test sessions [77]. Altogether, there were 64 trials per individual participant. When a fragment was completed with a correct keystroke, visual feedback was given comprising the completed fragment appearing in green for one second. When the keystroke was incorrect the correct word was displayed in red.

### Instruction

The materials used for instruction were similar to the word and picture slides used in the warm-up training. These same pictures were also used in the form of small cards for some games.

#### Overview of the training paradigm

The training paradigm consisted of activities aimed to encourage beginning learners to create multisensory mental representations of L2 constructions [78–80]. Learning activities were spread over several days to take advantage of spaced repetitions, to take advantage of testing effects, and because the content was deemed too difficult to learn in one day. Throughout the activities, learning engagement and motivation were supported in several ways:

- The words used to create the training and testing items were appealing, meaning they used words which were easy and generally well known, as ranked by young L2 German speakers of English in an unpublished study.
- The gestures used to create the training and testing items were deemed intuitive, as indicated by young L2 German speakers of English who viewed the gestures and marked on a list what they thought they had seen.
- Scaffolding was provided in such a way that teacher support faded over the sessions and encouraged a transfer of responsibility to the learners.
- Language games were played in different groups, some in pairs, some in small groups and some, such as class memory, were played all together.
- Discussion at the end of sessions allowed learners to reflect on what they learned and why it was important.

#### Gesture training

The gesture training was taught in one week and consisted of four 60 minute lessons spread over four days. In all lessons there was a balanced approach of direct instruction, modeling, guided practice and group games. The possessive and plural distinction was introduced in the second lesson through a sorting game modeled and played with the class with the item pictures projected at the front of the class room. After briefly explaining in German that an ‘s’ sound can ‘mean different things’ in English, (sometimes meaning more than one, and sometimes meaning that something belongs to or is a part of something else), a game was played where as a group children pointed to pictograms symbolizing the plural or possessive gesture. After this game was played with half the items the same game was repeated but this time instead of pointing the syntactically specific and plural and possessive gestures were used. Subsequently the other half of the items were treated in the same way. Most games involved in training took between 10 and 12 minutes to play. Lessons were conducted by the experimenter with the classroom teacher present who led the feedback sessions and replaced the regular English lessons.

### Procedures

#### Warm-up training procedures

In order to ensure that children were familiar with the words in the study, a warm-up training was conducted. This happened in two phrases. First, as a group learners were presented with a list of written words also containing two language-like words, *haque* and *adair* which follow the phonotactic rules of English but which are not English. After discussing which words were less familiar and revealing which words cannot be known (the pseudo-words), the 40 word-gesture pairs of the experiment were introduced in ‘word families’ or semantic fields. For example, to introduce the words in the baby semantic field (baby, crawl, smile, blanket and teddy) a slide at the front of the classroom projected the written word baby, and the experimenter demonstrated the baby gesture twice which was enacted by the children. The word-gesture demonstration was then followed by a picture-gesture demonstration before moving on to the next word. The 20 word-gesture pairs belonging to the *baby*, *boy*, *car* and *cat* semantic fields were introduced first and following a short break the remaining word-gestures pairs belonging to *dog*, *frog*, *girl* and *horse* followed.

#### Gesture speeded fragment testing procedures

Children participated individually at a table in a corner of an unused staff room at the school. Children were thanked for coming and, because of the pandemic, asked if they had washed their hands. They were then asked for their help in entering their ‘secret code’ which was the ID code used to match trials and language surveys. Children were seated in front of a laptop and after a brief explanation of why their hands needed a comfortable resting place in front of the keyboard, the first part of the task instructions were read in English: ‘You will see two words followed by a word with a missing letter. You decide which missing letter completes the word.’ Then the experimenter then demonstrated how to complete a sample fragment for a phrase not included in the task, *the cats j_mp* (jump). The second part of the instructions were then read in English and translated into the language of instruction, German. ‘To make things easier the answer will always be a, e, i, o, or u.’ Children were encouraged to find the letters on the laptop keyboard which were printed on the screen and would be used in the task before beginning the task. The task was self-paced and after the child began the task the experimenter moved to a nearby table so that the screen was not in direct sight.

After the first 32 items (between the two blocks) the children were asked if they would like to take a break. Children usually declined and helped once again to enter their ‘secret code’ and began the second block in the opposite condition. After completion each child was thanked and asked to notify the next child. The entire procedure usually took between 10–15 minutes per child. All fragments used in this task can be seen in S1 Appendix and additional details about the stimuli can be found in the Materials and Methods section.

### Data analysis

Multiple regression analyses were conducted on response time to test the effects of teaching using gestures which embody grammatical morphemes on procedural learning. Our continuous dependent variable (response time for word fragments) and our binary dependent variable (correct vs. incorrect responses for word fragments) were analyzed using a multilevel modeling approach. A hierarchical model including subject as a random effect. Session, meaning the time point when the tests were conducted, and condition were included as fixed effects. All analyses were conducted with R Version 4.0.3 with the *lme4* package [81].

## Results

### Data description

Our analysis of student outcomes employs a model comparison approach and includes students who completed both test sessions in both conditions. For each participant, a mean correct response time (i.e., mean response time for fragments correctly answered on the test) was calculated and responses which were slower than 2 SDs were removed. Erroneously completed targets comprised 17.9% of the data and response times slower than the individual cutoff value excluded another 3.8% of the data. PsychoPy did not accept responses faster than 250 ms and after applying the individual cutoff value no responses were slower than 5.7 seconds, so no further cleaning was necessary. This led to an average RT in the S (two-gesture) condition of 1.98 seconds (SD = .71) at pretest and 1.77 seconds (SD = .68) at posttest and an average RT in the G (one-gesture) condition of 1.94 seconds (SD = .75) at pretest and 1.84 seconds (SD = .71) at posttest.

### Differences between conditions

**Fig 3:**
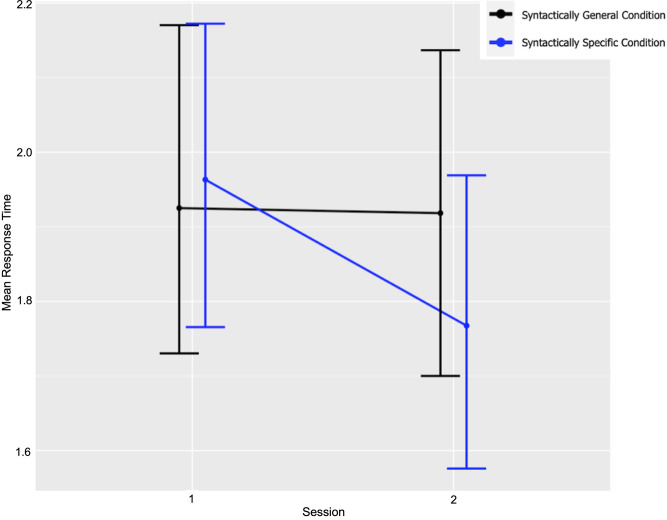
Change in mean response time for correct fragments between sessions by condition.

In Fig 3 the mean response times are plotted by session and condition. The confidence intervals are wide, reflecting the true uncertainty in the estimates of the means.

The *x-*axis plots the two tests, pretest (before instruction), post (one week after instruction), for the S (two-gesture) and G (one-gesture) conditions. The *y-*axis plots the mean response time for cleaned correct test items per teaching method. For the sake of clarity, error bars plot unadjusted 95% confidence intervals. However, this plot somewhat ignores the within-subjects design of the study. It does not tell us whether the observed decrease in RT for the syntactically specific condition over sessions is there because it occurred consistently for all subjects or because of a small number of subjects whose RT decreased very steeply. This can be checked by showing the plot separately by subject.

**Fig 4:**
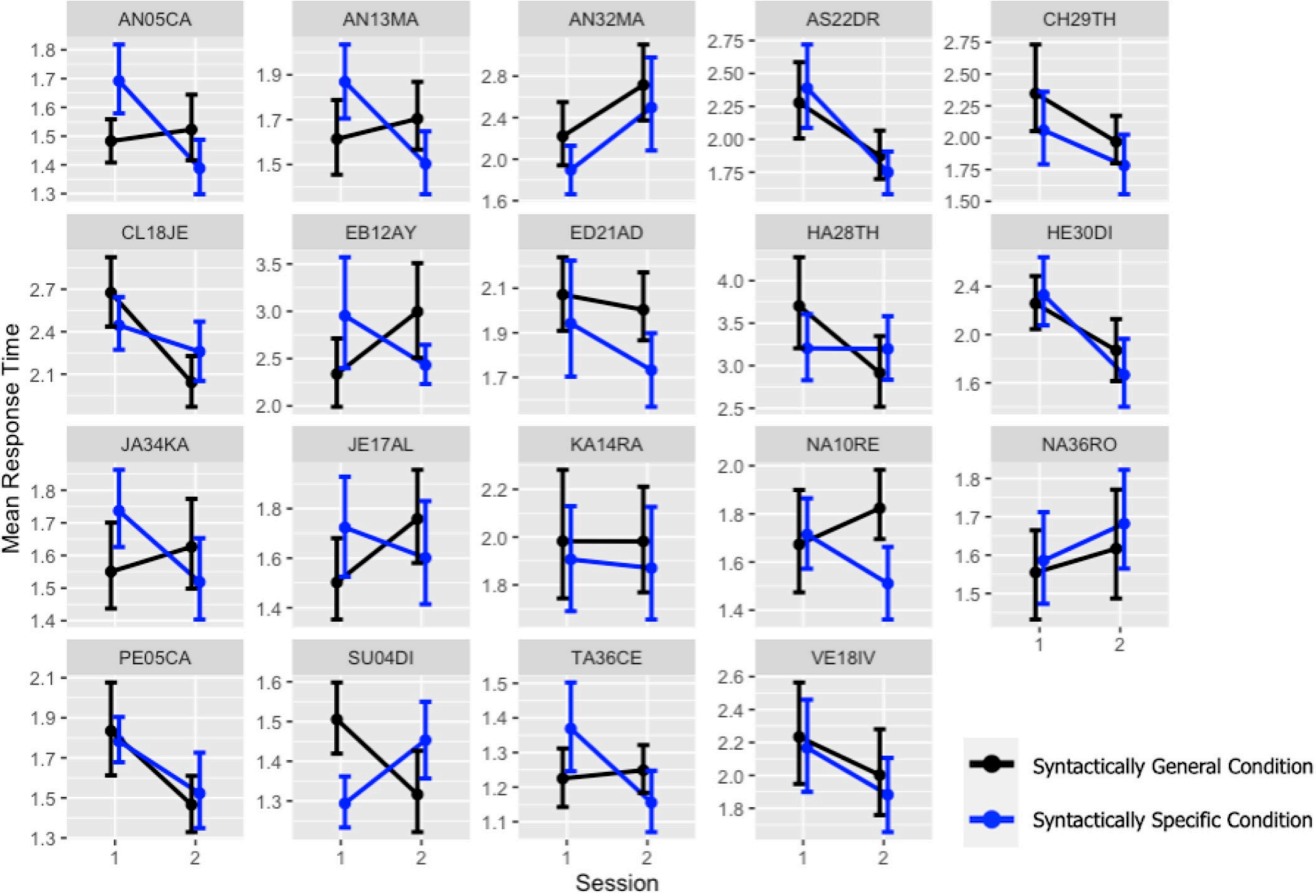
Change in mean response time between sessions and condition by participant.

Fig 4 shows children’s mean response time organized by session and condition. A fairly large number of subjects show a steeper session-to-session decrease in RT for condition S. But the pattern is not universal. Here again, the x-axis plots the two tests, once before instruction and once one week after instruction for both conditions. Again, the y-axis plots the mean response time for cleaned correct test items per teaching method and error bars plot unadjusted 95% confidence intervals.

### Long-term gain in procedural learning

In order to further investigate these differences, children’s response time was entered in a random effects model including subject as a random effect. Experimental session and condition were included as fixed effects. Four models were created: 1) a baseline model predicting RT with random intercepts and random slopes across subjects (mRandom); 2) a model with session as a predictor of RT and random intercepts across subjects (mRandom_session); 3) a model with session and condition as a predictors, and random intercepts across subjects (mRandom_condition); 4) a model with session and condition as predictors of RT, an interaction between session and condition, as well as random intercepts across subjects (mRandom_interaction) This incremental adding of terms is important. For example, without subjects, the first term added, no learning is possible. Without session, the second term added, it is not possible to measure a change in learning, as measured by a potential decrease in RT etc. Each time only one new component was added to the model in order to facilitate comparing them with the log-likelihood statistic.

**Fig 5:**
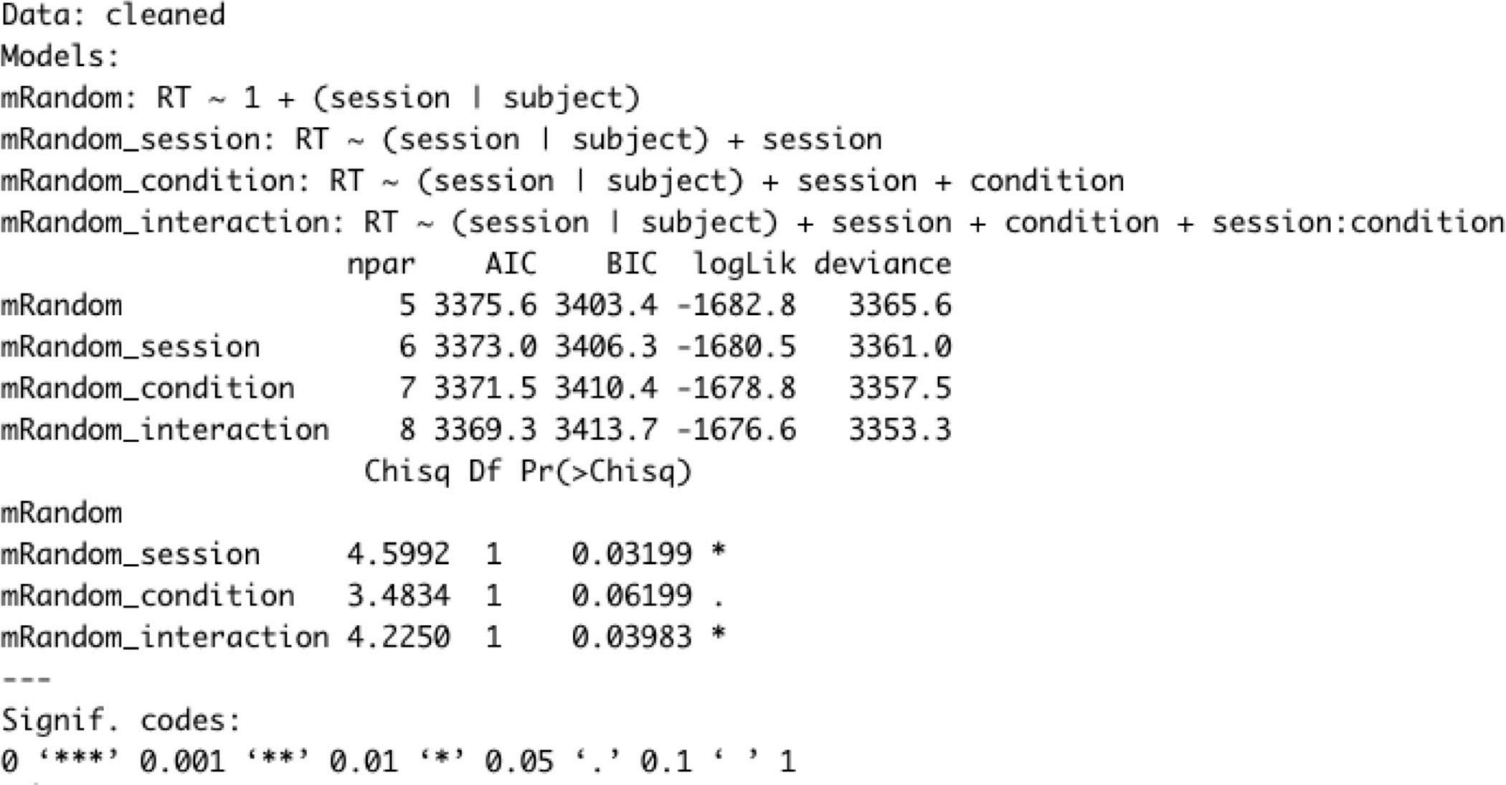
Random effects model output.

The resulting output seen in Fig 5 shows that adding session significantly improved the fit of the model, Chisq(1) = 4.59, p = .031. Adding the fixed effect of condition did not significantly improve the model, Chisq(1) = 3.48, p = .061. However, adding the interaction between session and condition did significantly improve model fit, Chisq(1) = 4.22, p = .039. A post hoc Tukey test showed that the S (two-gesture) and G (one-gesture) conditions differed significantly at p < .05.

Comparing the results of the two models summarized in Table 2 allows us to see that the fit of the model with the interaction between session and experimental condition is favored.

**Table 2:** Summary of model fit statistics.

	Df	AIC	BIC	log Lik	deviance	Chisq	Chi Df	P(>Chisq)
mRandom_condition	7	3371.5	3410.4	-1678.8	3357.5	3.48	1	.061
mRandom_interaction	8	3369.3	3413.7	-1676.6	3353.3	4.22	1	.039*

As can be seen from the output of the mRandom_condition model below, the interaction between the experimental group and session appears to be specific to the second time point or posttest in session 2. Based on Fig 3, this interaction is to be expected. Response times from the syntactically specific condition in the second testing sessions (p = .039 *) suggest that many learners are able to exploit the semantic information in the syntactically specific gestures enough to be measured by the GSF task.

### Summary

Visual inspection of Fig 3 suggests that a gain could be measured between pre and posttest in the syntactically specific condition. While this was not true for every individual learner, as can be seen in Fig 4, this change was statistically significant (p = .039) as expressed in the mRandom_interaction model output which includes an interaction between session and condition. These changes in response time show that there are large differences between learners. Especially for learning which is new, this result suggests that teaching over time is important in order to consolidate what has been learned [82]. In summary, the results for the final model (mRandom_interaction) are presented in Table 3.

**Table 3:** Summary of fixed and random effects.

				*Random effects*
	*Fixed effects*			By Subject
*Parameters*	Estimate	*SE*	*t value*	*SD*
Intercept	2.06	0.15	13.56	0.61
Session	–0.07	0.06	–1.25	-
Condition	0.11	0.08	1.38	-
Session x Condition	–0.10	0.05	–2.05	-

Model formula: mRandom_interaction: RT ~ (session | subject) + session + condition + session:condition.

## Discussion

Through using a novel task, the gesture speeded fragment completion task, this study sought to investigate the effectiveness of L2 teaching which visually differentiates between grammatical morphemes which differ in meaning but sound the same. Essentially, this training focused on encouraging children to connect their sensorimotor experiences (viewing and performing speech and gesture combinations in a group) to explicit information related to phrases containing the plural{-s} and 3rd person possessive {-s} in English. Both this embodied approach and the fact that particular attention was devoted to mentally simulating phrases containing grammatical morphemes which differ in meaning but sound the same provide an advance over prior empirical work [60,61]. Also, it moves beyond current classroom practices on L2 instruction where learning as a multisensory experience has so far hardly pervaded [32]. Regarding training and procedural learning, the experimental results provide the following valuable insights.

The main finding of this study is that under authentic teaching and learning conditions, the gesture training decreased the mean response time in the children’s fragment completion performance. Specifically, following the gesture-based training, most grade-six children showed a larger pretest-to-posttest improvement on the gesture speeded fragment completion task, our test of procedural learning, in the syntactically specific (two-gesture) condition than in the syntactically general (one-gesture) condition. Given that the phrases (and fragments) in both conditions were the same, it is unlikely that sixth graders’ improved performance in the syntactically specific condition is simply the result of faster fragment completion in the posttest. Rather, considering the activities the training actually encompassed, this finding suggests that children were able to use the additional information in the syntactically specific gestures for semantic prediction resulting in a greater decrease in word fragment response time in the two-gesture test condition.

Our study demonstrates that the aggregate of instructions and exercises encouraging these children to connect words and then noun and verb phrases to their sensorimotor experiences improved their fragment completion performance. It remains to be explored in future research to what extent the gestural benefit observed in this study can be generalized to other syntactic learning situations. Also, it is yet unclear why the gesture-based training was more effective for some children than for others. Additional studies with different paradigms and more participants are required to investigate this question. Nonetheless, the changes in response time over time raise certain questions worth investigating. Before addressing one additional question, I would like to addresses the original research questions:

1. In the context of a gesture-based training, can a long-term gain in L2 procedural learning for the use of grammatical morphemes be measured on a transfer task?
2. If the same test items are used in both conditions, does seeing different grammatical morphemes in gesture form (syntactically specific vs syntactically general gestures) result in measurable differences in response time?

Regarding question one, visual inspection of Fig 3 shows a gain in procedural learning between pre and posttest. Moving on to question two, this gain is found in the syntactically specific two-gesture condition. This is confirmed by the model output which includes an interaction between test session and test condition (p = .039*). As can be seen in Fig 4, this was not true for all learners, however, what this exactly means is not easy to interpret. Learning is complex and there are many interactions between procedural and declarative learning processes. For example, following Ferman and Karni [59], in this experiment a decrease in response time was evaluated as an increase in procedural learning. However for a few learners, (presumably those with a lower level of L2 ability) it is possible that because the gesture-based training highlighting the meaning of plural and possessive grammatical morphemes, more attention and awareness (not less), could have resulted in an increase in response time for completing fragments. Ferman and Karni [41] write: “There is evidence … that as a result of training and experience, implicit knowledge can become explicit in the sense that learners can become aware of the underlying structures and regularities (rules).” For other learners, (presumably those with a higher level of L2 ability) this process of becoming aware of grammatical rules could also be associated with a temporary decrease in speed. Hebbian mechanisms for synaptic modification explain why consolidation of learning is an important concept and insufficient consolidation could provide a rationale why learning from four lessons of instruction did not increase procedural learning for all children. A follow-up experiment could space teaching over several weeks, as opposed to just one. In addition to spaced teaching, an experiment which addresses interaction effects between gesture-based instruction and L2 writing would be of interest.

The additional question I would like to ask is if the GSF task may have been too complex. In order to collect response time the task needs to go through spelling, then the word, and then the concept. This means that a knowledge of spelling is needed to access the concept and syntactic learning. On the other hand, although some children struggle with L2 writing and spelling, these skills are taught and are required for academic success, and exit interviews from testing consistently confirmed that children enjoyed the challenge of the ‘game’.

## Conclusion

This experiment uses the gesture speeded word fragment completion task and asks if learners observing syntactically specific L2 ‘s’ gestures which visually distinguish between the plural and possessive ‘s’ enhance linguistic processing in comparison to a single ‘s’ gesture which does not make this distinction. It is well-established that gestures support L2 word learning, however research on the effect of gestures on syntax is rare. As a teaching tool, gestures are easily accessible and can be paired with different linguistic units. However, if there is no difference between exposing L2 learners to gestures which are syntactically general or syntactically specific, this would suggest that language teachers should not support learners by using gesture systems which make this distinction. Efficient language learning processes are key in multilingual societies and understanding when and how gesture promotes learning can help put this important teaching and learning tool to optimal use.

In conclusion, recent decades have witnessed an increase in interest in the roles of embodied teaching methods, but there is still a need for more empirical work that explores the results of student and teacher gestures in naturalistic classroom interactions. This is particularly the case for contexts of L2 teaching beyond investigating vocabulary learning. By combining gesture theory and research from the classroom, this paper provides evidence that gestures can promote procedural knowledge for difficult L2 morpho-syntactic structures, such as the English plural{-s} and 3rd person possessive{-s} in primary school. Importantly, our findings suggest that for sixth-grade children, the same verbal information can be packaged in different ways and that these nuanced differences may have important implications for teaching and learning syntax. Rather than just supporting learners to understand a grammatical rule, it is important to use teaching methods which encourage enactments of sensorimotor experiences [83]. More research is certainly needed to further develop and refine such an approach. The transfer of concept learning from perceiving gestures in a social setting to solving a written task is in line with research that shows that neglecting movement as a learning strategy leaves an important source of support under-utilized [80]. This present study not only serves as a useful starting point from which future endeavors can be explored, it also suggests that this would provide a valuable addition to L2 instruction.

## Supporting information

S1 Appendix Items used in training and testing. (DOCX)
