# Supplementary material for: Can grammatical morphemes be taught? Evidence of gestures influencing second language procedural learning in middle childhood
Source: PLoS One. 2023 Feb 1;18(2):e0280543. doi: 10.1371/journal.pone.0280543 (PMC9891517; doi:10.1371/journal.pone.0280543)
Supplement: S1 Appendix — (DOCX) [file pone.0280543.s001.docx]

1. **S1 Appendix**
2. Items used in training and testing

| the babies crawl the babies smile the baby's blanket the baby's teddy | the dogs bark the dogs play the dog's neck the dog's tooth |
| --- | --- |
| the boys point the boys wave the boy's book the boy's t-shirt | the frogs jump the frogs listen the frog's foot the frog's tongue |
| the cars crash the cars move the car's window the car's wheel | the girls hug the girls whistle the girl's dress the girl's hair |
| the cars crash the cars move the car's window the car's wheel | the girls hug the girls whistle the girl's dress the girl's hair |
| the cats sleep the cats look the cat's nose the cat's tail | the horses kick the horses walk the horse's head the horse's mouth |
